# Repeated Autologous Bone Marrow Transfusion through Portal Vein for Treating Decompensated Liver Cirrhosis after Splenectomy

**DOI:** 10.1155/2018/4136082

**Published:** 2018-10-29

**Authors:** Weiwei Zhang, Mujian Teng, Baochi Liu, Qiling Liu, Xin Liu, Yanhui Si, Lei Li

**Affiliations:** ^1^Department of Hepatobiliary Surgery, Qianfoshan Hospital Affiliated to Shandong University, Jinan, China; ^2^Department of General Surgery, Shanghai Public Health Clinical Center, Fudan University, Shanghai, China

## Abstract

**Objective:**

This study is aimed at examining the impact of repeated intraportal autologous bone marrow transfusion (ABMT) in patients with decompensated liver cirrhosis after splenectomy.

**Methods:**

A total of 25 patients with decompensated liver cirrhosis undergoing splenectomy were divided into ABMT and control groups. The portal vein was cannulated intraoperatively using Celsite Implantofix through the right gastroomental vein. Both groups were given a routine medical treatment. Then, 18 mL of autologous bone marrow was transfused through the port in the patients of the ABMT group 1 week, 1 month, and 3 months after laminectomy, while nothing was given to the control group. All patients were monitored for adverse events. Liver function tests, including serum albumin (ALB), alanine aminotransferase (ALT), total bilirubin (TB), prothrombin activity (PTA), cholinesterase (CHE), *α*-fetoprotein (AFP), and liver stiffness measurement (LSM), were conducted before surgery and 1, 3, and 6 months after surgery.

**Results:**

Significant improvements in ALB, ALT, and CHE levels and decreased LSM were observed in the ABMT group compared with those in the control group (*P* < 0.05). TB and PTA improved in both groups but with no significant differences between the groups. No significant changes were observed in AFP in the control group, but it decreased in the ABMT group. No major adverse effects were noted during the follow-up period in the patients of either group.

**Conclusions:**

Repeated intraportal ABMT was clinically safe, and liver function of patients significantly improved. Therefore, this therapy has the potential to treat patients with decompensated liver cirrhosis after splenectomy. This trial was registered with the identification number of ChiCTR-ONC-17012592.

## 1. Introduction

Liver cirrhosis is an advanced-stage liver disease representing irreversible damage or scarring to the liver and is a major cause of mortality worldwide [1]. Liver transplantation is so far the only effective treatment for decompensated cirrhosis; however, its application is largely restricted by technical difficulties and limited donor sources [2]. Recent advances in stem cell research have prompted the development of stem cell-based therapy in these patients since the stem cells, including bone marrow-derived stem cells, have the capacity for self-renewal and multilineage differentiation [3]. Positive results from stem cell therapy using animal models have led to the evaluation of the feasibility and safety of bone marrow cell (BMC) therapy in patients with chronic liver disease [4–8]. As an accessible source of stem cells, adult human bone marrow contains two major types of stem cells: hematopoietic stem cells (HSCs) and mesenchymal stromal cells (MSCs). HSCs are capable of both self-renewal and differentiation into multiple hematopoietic lineages, while MSCs are nonhematopoietic and represent a minute fraction (0.001%–0.01%) of the total nucleated cell population in the marrow [9].

Previous studies have also shown that transplantation of bone marrow-derived mesenchymal stem cell (BMSC) effectively reduced liver fibrosis and improved liver function by inducing regeneration of the liver in different animal models [10] through the paracrine action of BMSC and fusion with hepatocytes [11, 12]. Nevertheless, cells undergo extensive *in vitro* expansion to obtain a sufficient number of MSCs prior to transplant, increasing the risk for genetic mutations and eventually leading to malignant transformation [13] and decrease of the adipogenic and osteogenic differentiation potential in long-term expanded MSCs [14]. Homing of culture-expanded MSCs is inefficient compared with leukocytes and HSCs due to a lack of relevant cell adhesion and chemokine receptors [15].

It was hypothesized in this study that bone marrow transfusion would improve homing of MSCs and repeated bone marrow transfusion would result in more sustained clinical efficacy and improved liver functions. This exploratory study was therefore designed to investigate the safety and feasibility of the repeated bone marrow transfusion treatment in patients with decompensated liver cirrhosis.

## 2. Materials and Methods

### 2.1. Patients

A total of 25 patients with decompensated liver cirrhosis were recruited for this study. All patients attended the Shanghai Public Health Clinical Center, China, from November 2014 to December 2015 and were diagnosed with decompensated liver cirrhosis caused by chronic hepatitis B, hepatitis C, alcoholic cirrhosis, and schistosomiasis cirrhosis. This study was approved by the ethics committee of the Shanghai Public Health Clinical Center, Shanghai, China. This trial was registered with the identification number of ChiCTR-ONC-17012592. All patients provided written informed consent. Patients required splenectomy because of portal hypertension or hypersplenism and met all the following inclusion criteria: age 20–70 years, decompensated liver cirrhosis, abnormal serum albumin (ALB) and/or bilirubin and/or prothrombin time, and no viable hepatocellular carcinoma seen on a computed tomography (CT) scan. The exclusion criteria were evidence of extrahepatic biliary diseases, severe cardiac insufficiency, and no severe diseases such as stroke or myocardial infarction in the last 6 months. All patients were subjected to splenectomy which was performed by laparotomy and associated with nonselective esophagogastric devascularization. During splenectomy, the Celsite Implantofix (B. Braun Medical Inc., France) was implanted in the portal vein through the right gastroomental vein. After surgery, the patients were assigned into two groups: patients who received treatment of autologous bone marrow transfusion (ABMT) and patients who were given nothing (control).

### 2.2. Bone Marrow Transfusion

Before treatment, all patients underwent upper abdominal vascular enhanced CT examinations to identify the spatial relationship of the portal veins and the hepatic veins and to exclude the possibility of liver tumors. One week after surgery, 36 mL of bone marrow was extracted from the anterior superior iliac spine using a syringe containing heparin in the ABMT group. Then, 18 mL of bone marrow was transfused into the portal vein using Celsite Implantofix in the ABMT group, while nothing was given to the control group. The other 18 mL was analyzed for cell number count, viability, and endotoxin. One month and 3 months after surgery, 18 mL of bone marrow was extracted and transfused directly without analyzed. The stem cell surface markers HLA-DR, CD34, CD45, CD31, CD14, CD19, CD71, CD105, CD73, and CD90 were quantified using appropriate antibodies (Becton Dickinson and Co., NJ, USA) on a fluorescence-activated cell sorting system Gallios (Beckman Coulter, USA).

### 2.3. Follow-Up

**Figure 1:**
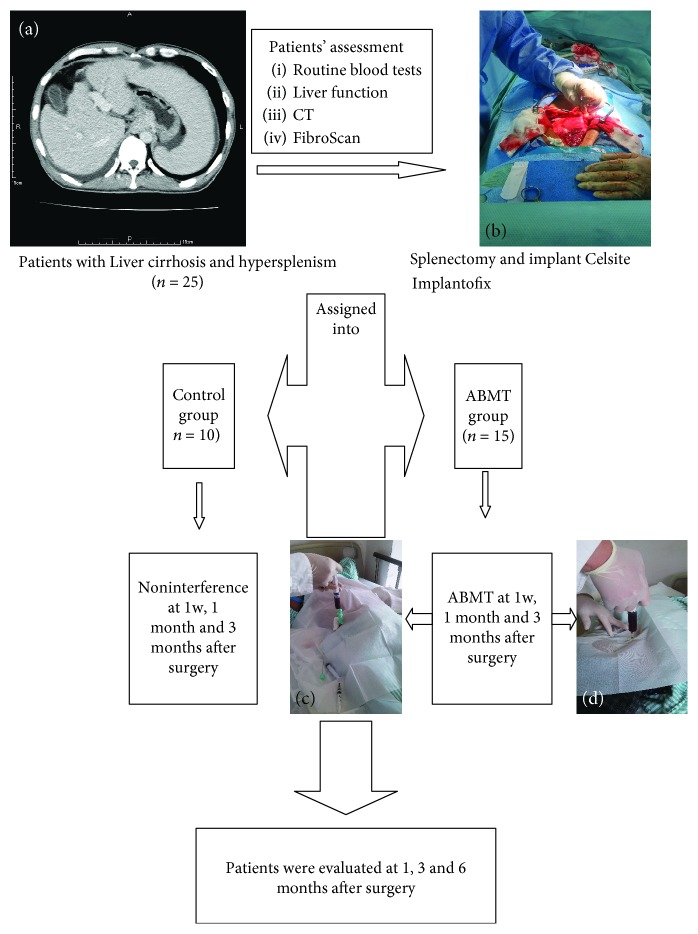
Flowchart of the study design. Representative images during the procedure are shown: (a) CT manifestations in patients with liver cirrhosis (*n* = 25), (b) splenectomy and implant using Celsite Implantofix (*n* = 25), (c) bone marrow extraction from the anterior superior iliac spine (*n* = 15), (d) ABMT (*n* = 15).

The flowchart of the study design is shown in Figure 1. Briefly, patients were evaluated 1, 3, and 6 months after surgery. Functional liver indices, including ALB, alanine aminotransferase (ALT), total bilirubin (TB), prothrombin activity (PTA), cholinesterase (CHE), and *α*-fetoprotein (AFP), were used to evaluate the overall condition of patients with decompensated liver cirrhosis. Patients were examined using FibroScan to evaluate liver stiffness measurement (LSM). Treatment effectiveness was defined as a decline in LSM after treatment. The evaluation of liver function ended 6 months after surgery. Adverse effects, such as fever, rash, and nausea, were recorded. Patients' medications except antiviral therapy were unchanged before and after the therapy and throughout the postoperative follow-up period (except for dosage reduction as a result of improvement in a patient's condition).

### 2.4. Statistical Analyses

SPSS for Windows version 17.0 software package (SPSS Inc., IL, USA) was used for statistical data analysis. Descriptive statistics were presented as mean ± standard deviation for quantitative variables, whereas qualitative data were expressed as numbers (frequency) and percentages. The changes in liver function parameters relative to baseline 1, 3, and 6 months after treatment were analyzed by multifactor analysis of variance for repeated measures. The chi-square or Fisher's exact test was used for qualitative data. A *P* value < 0.05 was considered statistically significant. The data used to support the findings of this study are included within this article.

## 3. Results

### 3.1. Patient Characteristics

A total of 25 patients (17 men and 8 women) with a mean age of 47.2 ± 10.21 years (range, 28–62) were enrolled in this study and distributed into the control (*n* = 10) and ABMT (*n* = 15) groups. Of the 25 patients included, 22 had hepatitis B cirrhosis, 1 had hepatitis C cirrhosis, 1 had alcoholic cirrhosis, and 1 had schistosomiasis cirrhosis. Group division is shown in Table 1. Demographic and clinical characteristics of the patients are presented in Table 2. At baseline, no significant differences were found between the two groups with regard to age, sex, and levels of ALT, ALB, CHE, TB, PTA, routine blood test (white blood cell (WBC), red blood cell, and platelet (PLT)), LSM, Child–Pugh, and AFP.

### 3.2. Isolation and Characterization of BMCs

**Figure 2:**
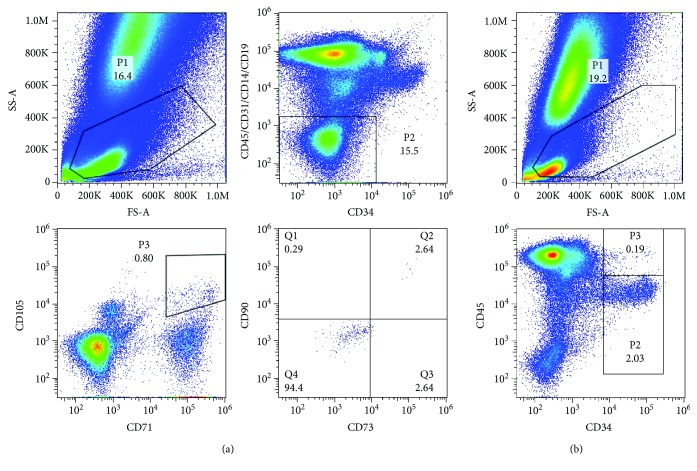
Representative cell surface antigen expression profile of isolated BMCs analyzed by flow cytometry: (a) MSCs, (b) HSCs.

The characteristics of the BMCs isolated from the patients are shown in Figure 2. Further, 18 mL of autologous bone marrow in which the mean number of infused MSCs was 1183.75 ± 493.6 and the mean number of infused HSCs was 712362.5 ± 493434.14 was transfused in the patients of the ABMT group. The relative percentage of various BM cell populations is shown in Table 3. The isolated BMCs from all patients had a cell viability > 95%. All samples were negative for endotoxin. Isolated MSCs were CD45^−^, CD31^−^, CD14^−^, CD19^−^, CD34^−^, CD105^+^, CD71^+^, CD73^+^, and CD90^+^. Isolated HSCs were CD45^−^ and CD34^+^.

### 3.3. Liver Biochemical Parameters

**Figure 3:**
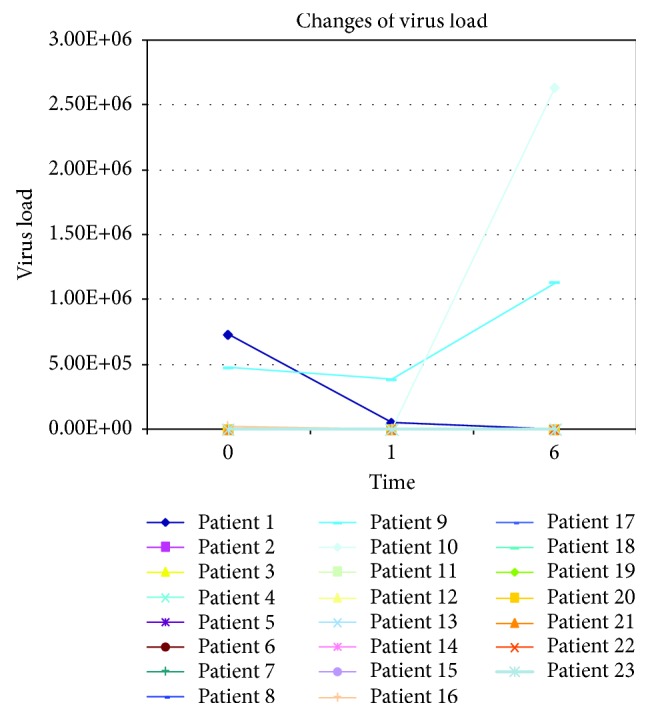
Changes of virus load of patients with decompensated liver cirrhosis caused by chronic hepatitis B and hepatitis C. Patient 9 was with hepatitis C. Patient 1 belonged to the control group, and patient 15 belonged to the AMBT group; the lamivudine had been replaced by entecavir before surgery. Patient 10 belonged to the AMBT group; the lamivudine had been replaced by entecavir in 6 months after surgery. The virus load of the rest of the patients is stable.

All patients were followed up for 6 months. All patients with hepatitis B have been treated with antiviral therapy (lamivudine, telbivudine, adefovir dipivoxil, and entecavir) except for one patient who was anti-HBs antibody positive. The patients with hepatitis C have not been treated with antiviral drugs because the antiviral drugs have not been approved by China Food and Drug Administration. In addition to the increase of the viral load in individual patients because of the cause of drug resistance, most of the patients' viral loads are stable. After the increase of the viral load in 3 patients treated by lamivudine, the lamivudine had been replaced by entecavir. Then, the viral load was negative. The changes of the viral load are shown in Figure 3.

**Figure 4:**
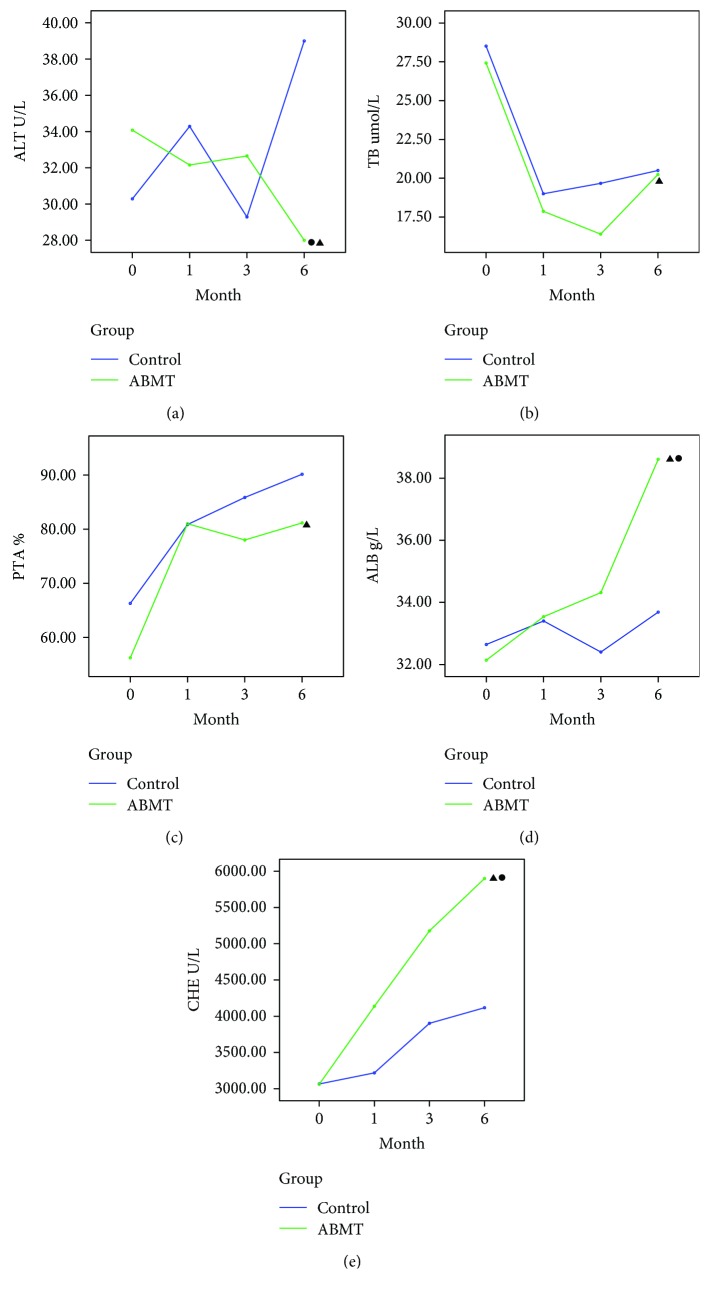
Changes in biochemical parameters after splenectomy or combined splenectomy and ABMT therapy in patients with decompensated liver cirrhosis: (a) ALT, (b) TB, (c) PTA, (d) ALB, and (e) CHE. ^▲^*P* < 0.05 indicates a significant difference versus the baseline value. ^●^*P* < 0.05 indicates a significant difference between ABMT and control groups.

The changes in functional liver indices, including ALB, ALT, TB, PTA, and CHE, are shown in Figure 4. In the ABMT group, the LSM decreased effectively and the total effective rate was 80%. In the control group, the total effective rate was 20%. A statistically significant difference was found in the total effective rate between the two groups (*P* < 0.05). The ALT levels in the ABMT group significantly decreased at 6 months, while it increased in the control group. A statistically significant difference was found between the two groups at 6 months (39 ± 27.03 U/L vs. 28 ± 9.22 U/L, *P* < 0.05). The TB levels in the two groups significantly decreased in 6 months but with no statistically significant difference between the two groups (20.5 ± 7.02 vs. 20.24 ± 6.36 mmol/L, *P* > 0.05). The PTA levels greatly increased in both groups after 6 months of treatment relative to baseline. No statistically significant difference was found between the two groups 6 months after treatment (90.14 ± 9.34% vs. 81.15 ± 11.68%, *P* > 0.05). The ALB levels greatly increased in the ABMT group after 6 months of treatment relative to baseline. The mean ALB concentration for patients in the ABMT group was 38.61 ± 4.05 g/L 6 months after treatment versus 33.54 ± 3.37 g/L before treatment (*P* < 0.05). No significant increase was found in the ALB levels in the control group compared with the preoperative levels. A statistically significant difference was found between the two groups (*P* < 0.05) at 6 months. The CHE levels greatly increased in both groups 6 months after treatment relative to baseline and significantly increased in the ABMT group compared with the control group; the difference between the two groups was statistically significant (*P* < 0.05).

### 3.4. Adverse Effects and Complications

After ABMT into the portal vein using Celsite Implantofix, no bleeding or thrombotic episodes were observed. The AFP levels of the control group had no significant change. However, the AFP levels in the ABMT group decreased. No other adverse effects (such as infection or hypersensitivity) were observed in any of the patients during or after the bone marrow transfusion.

## 4. Discussion

Patients receiving ABMT demonstrated considerable improvement in their laboratory data, with no procedure-related complications [16]. It is important to treat the causative liver disease, such as viral hepatitis, to maintain the effect of such therapies and enhance the resistance of infused cells to viral infection [17] or repeat the injection of stem cells at intervals to achieve sustained response [18].

The ability to isolate the subset of marrow stromal cells with the most extensive replication and differentiation potential would naturally be of utmost importance for both theoretical and applicative reasons. However, identifying the “phenotypic fingerprint” of a stromal stem cell may well be like shooting at a moving target, in that they seem to be constantly changing in response to their microenvironment, both *in vitro* and *in vivo* [19]. Noncultured autologous whole bone marrow was used in this study for the clinical application of liver regeneration therapy. First, no teratoma formation was observed, as seen with embryonic stem cells. Second, these cells were easier to collect compared with the hepatic stem cells in the liver. Moreover, the use of autologous BMC eliminated the problem of potential immunological rejection. With regard to the whole bone marrow, marrow pericytes might be heterogeneous. Some might be recruited during blood vessel formation from resident, preexisting osteogenic cells; others might originate from endothelial cells, and still others might grow from preexisting pericytes during vascular growth. Some would be osteogenic in nature, while others would not [19]. Liver regeneration requires not only hepatic stem cells but also other stromal cells. Therefore, it was proposed that the transplant should be “seeds with the soil,” that is, the whole bone marrow transplantation.

Terai et al. have recently shown [8] significant increases in the ALB and total protein levels, an improvement in the Child–Pugh score in all nine patients, and reductions in ascites in six patients with liver cirrhosis after autologous BMC infusion therapy. Gordon et al. [5] also showed that the injection of CD34^+^ cells into either the portal vein or the hepatic artery improved liver function in patients with liver insufficiency. Noncultured autologous whole bone marrow transfusion (18 mL) was evaluated three times in the present study for treating decompensated liver cirrhosis. 10 patients were treated with nothing and 15 with the bone marrow transfusion. Significant improvements in serum levels of ALT, TB, ALB, PTA, and CHE were observed in patients treated with ABMT. A statistically significant difference was found in ALT, ALB, and CHE between the control and ABMT groups throughout the follow-up period.

Previous studies have shown that splenectomy could improve the liver function. WBC and PLT significantly increased after splenectomy. Serum levels of TB and prothrombin time significantly improved after splenectomy [20]. Significant improvements in the serum levels of ALB, PTA, and CHE were observed in the control group in the present study.

Sandrin et al. [21] had shown that liver stiffness measurements (LSMs) were reproducible, operator-independent and well correlated to fibrosis grade (METAVIR). It is believed that the activation of hepatic stellate cells or myofibroblasts and portal myofibroblasts in the liver leads to the formation and deposition of extracellular matrix, which is the main mechanism of hepatic fibrosis. Oyagi et al. [22] had found that the transplantation of the bone marrow-derived mesenchymal cells (BMMCs) into liver-injured rats significantly suppressed liver fibrosis. In our study, in the ABMT group, the LSM decreased effectively (12 in 15) and the total effective rate was 80%. In the control group, 2 patients were detected with a decrease in LSM in 10 patients. The total effective rate was 20%.

Several possible routes exist for BMC administration, including through the peripheral vein, hepatic artery, or portal vein [8, 23–25]. Celsite Implantofix was implanted in this study in the portal vein through the right gastroomental vein. This method was as simple as the peripheral intravenous infusion, causing almost no risk of operation (such as bleeding and infection); the cost was also relatively low compared with traditional treatment. More importantly, it was much easier to handle than the direct BMC liver transplantation. Further, it was extremely convenient to carry out repeatedly, thereby solving the problem of single BMC transfusion insufficiency.

None of the patients had adverse reactions to ABMT in the present study. The AFP level of two patients in the ABMT group was 435.7 and 149 ng/mL, respectively. No liver cancer was found. The AFP levels decreased after treatment. Moreover, hematopoietic or solid tumors did not develop in the follow-up period of 6 months in the ABMT group.

A small amount of autologous bone marrow was transfused in this study because of the uncertainty in the safety of ABMT and the patient's tolerance of the volume of the extracted bone marrow. However, autologous bone marrow was transfused three times at intervals to achieve a sustained response. The relationship between the volume of the bone marrow transfused and the curative effect will be explored in future studies.

## 5. Conclusions

In conclusion, repeated autologous whole bone marrow transfusion therapy is promising because it improved the liver function of patients with liver cirrhosis. A larger-scale, randomized, double-blinded study is necessary to demonstrate the full therapeutic value of this protocol. Moreover, the present study was limited by the different etiologies of cirrhosis and baseline patient heterogeneity. Further studies with higher numbers of patients are warranted to better clarify the effect of autologous whole bone marrow transfusion on cirrhosis.

## Figures and Tables

**Table 1:** Group division.

	Control (*n* = 10)	ABMT (*n* = 15)
Chronic hepatitis B	8	14
Chronic hepatitis C	0	1
Alcoholic cirrhosis	1	0
Schistosomiasis cirrhosis	1	0

**Table 2:** Clinical characteristics of patients.

	Groups		*P* value
	Control	ABMT	
Sex (male, %)	80%	60%	>0.05
Age (year)	51.29 ± 11.38	45 ± 9.23	>0.05
ALT (U/L)	30.29 ± 15.84	32.42 ± 21.41	>0.05
TB (*μ*mol/L)	28.51 ± 11.91	27.42 ± 20.37	>0.05
PTA (%)	66.29 ± 9.14	56.23 ± 11.27	>0.05
ALB (g/L)	32.64 ± 5.2	32.14 ± 5.46	>0.05
CHE (U/L)	3065 ± 1661	3060 ± 847.9	>0.05
WBC (10^9^/L)	2.27 ± 0.71	2.27 ± 1.60	>0.05
RBC (10^9^/L)	3.66 ± 0.72	3.19 ± 0.55	>0.05
PLT (10^9^/L)	43.11 ± 24.11	44.36 ± 22.01	>0.05
Child–Pugh	7.44 ± 1.23	7.71 ± 1.48	>0.05
LSM (kPa)	22.25 ± 12.78	22.06 ± 13.29	>0.05
AFP (ng/mL)	3.4 ± 2.66	54.68 ± 122.35	>0.05

AFP: *α*-fetoprotein; ALB: serum albumin; ALT: alanine aminotransferase; CHE: cholinesterase; LSM: liver stiffness measurement; PLT: platelet; PTA: prothrombin activity; RBC: red blood cell; TB: total bilirubin; WBC: white blood cell. Values are mean ± standard deviation.

**Table 3:** The relative percentage of various BM cell populations.

	Statistics %	Cells (18 mL bone marrow)
Hematopoietic stem cells (HSCs)	0.32 ± 0.20	712362.5 ± 493434.1
Mesenchymal stem cells (MSCs)	0.00051 ± 0.00014	1183.75 ± 493.6

## Data Availability

The data used to support the findings of this study are available from the corresponding author upon request.
